# Mechanisms of social buffering of fear in zebrafish

**DOI:** 10.1038/srep44329

**Published:** 2017-03-31

**Authors:** Ana I. Faustino, André Tacão-Monteiro, Rui F. Oliveira

**Affiliations:** 1 Instituto Gulbenkian de Ciência, Rua da Quinta Grande 6, Oeiras 2780-156, Portugal; 2 ISPA–Instituto Universitário, Rua Jardim do Tabaco 34, Lisboa 1149-041, Portugal; 3 Champalimaud Neuroscience Programme, Champalimaud Centre for the Unknown, Avenida Brasília, Lisboa 1400-038, Portugal

## Abstract

Some humans thrive whereas others resign when exposed to threatening situations throughout life. Social support has been identified as an important modulator of these discrepancies in human behaviour, and other social animals also exhibit phenomena in which individuals recover better from aversive events when conspecifics are present – aka social buffering. Here we studied social buffering in zebrafish, by exposing focal fish to an aversive stimulus (alarm substance – AS) either in the absence or presence of conspecific cues. When exposed to AS in the presence of both olfactory (shoal water) and visual (sight of shoal) conspecific cues, focal fish exhibited a lower fear response than when tested alone, demonstrating social buffering in zebrafish. When separately testing each cue’s effectiveness, we verified that the visual cue was more effective than the olfactory in reducing freezing in a persistent threat scenario. Finally, we verified that social buffering was independent of shoal size and coincided with a distinct pattern of co-activation of brain regions known to be involved in mammalian social buffering. Thus, this study suggests a shared evolutionary origin for social buffering in vertebrates, bringing new evidence on the behavioural, sensory and neural mechanisms underlying this phenomenon.

The ubiquity of group formation among animals has been explained by its anti-predatory advantages, including an overall increase in vigilance (many-eyes hypothesis), the dilution of risk (selfish herd hypothesis) and predator confusion^1^. Accordingly, social groups offer a safer environment in the presence of threat and the presence of conspecifics is known to down-regulate the response to a detected threatening event, a phenomenon named social buffering (SB)^2^,^3^,^4^.

This social phenomenon has been documented in mammals, where there is already some evidence about its neural mechanisms^4^,^5^,^6^, but its study in other vertebrate taxa is still scarce (e.g. birds^3^). Thus, comparative studies are needed to better understand the evolution of SB among social animals and how evolutionary conserved are its underlying mechanisms. Moreover, different sensory modalities can convey relevant social cues used in the buffering behaviour^7^,^8^,^9^. Therefore, a single SB episode may have the contribution of distinct sensory cues (e.g. olfactory and visual) and the effectiveness of different sensory modalities to the same buffering event may vary. Consequently, we suggest that understanding the effectiveness of distinct sensory cues to the buffering behaviour is of particular relevance, considering that it directly influences the individual’s chances of survival. Also, establishing a robust sensory stimulus has been one of the crucial aspects for assessing the neural mechanisms regulating behaviour^10^. Therefore, determining the efficiency of different sensory modalities for a given SB event will enable the accurate dissection of the neural basis of this social behaviour.

Regarding the neural mechanisms underlying SB, a few studies in mammals have shown that the reduced fear behaviour (freezing) in the presence of conspecific cues is accompanied by a lower activation of brain areas such as the paraventricular nucleus of the hypothalamus – PVN; the lateral amygdala – LA; and the central amygdala – CeA^2^,^6^,^8^,^9^. However, the neural basis of the buffering behaviour has been poorly investigated, and studies that allow a further understanding of the neural processing of this phenomenon are still missing. Given their phylogenetic position, teleost fish offer the possibility to investigate the occurrence of SB and its neural mechanisms in the most successful evolutionary radiation among vertebrates, parallel to that of tetrapods^11^. Among teleosts, zebrafish offer a unique opportunity for such studies since they: (a) live in structured social groups^12^,^13^; (b) exhibit a stereotypic fear-like alarm response that has been well characterized^14^,^15^; (c) respond to both olfactory and visual cues of fear by conspecifics^16^,^17^; (d) are a model organism, with neurogenetic tools available to study the neurobiological basis of fear^18^,^19^,^20^ and social behaviour^21^,^22^,^23^,^24^; and (e) present homologous brain areas to those of mammals^25^,^26^,^27^,^28^,^29^.

Here, we developed a behavioural paradigm to investigate SB in zebrafish when exposed to AS, which is a known fear-eliciting stimulus in this species^14^,^15^. In order to test SB, fish were exposed to AS in the absence or presence of conspecific cues (olfactory and/or visual) and their fear response was quantified. Since different sensory modalities are known to contribute to SB^7^,^8^,^9^, we then assessed the individual contribution of olfactory and visual cues to SB, and explored their separate effectiveness in acute and long-lasting exposures to threat. Subsequently, we investigated the influence of shoal size on the buffering phenomenon, as different shoal sizes are known to distinctly modulate responses to threatening events^30^,^31^. Finally, in order to explore the neural mechanisms underlying SB in zebrafish, we investigated the activation of brain regions known to be involved in SB^2^,^4^,^6^,^8^,^9^, fear^32^,^33^,^34^,^35^, anxiety^35^,^36^,^37^ and social behaviour regulation^38^,^39^,^40^,^41^ in other species. In sum, our study suggests a shared evolutionary origin for SB in vertebrates and establishes zebrafish as a genetically tractable vertebrate model system for the study of the buffering phenomenon at the behavioural and mechanistic level, focusing on the sensory and neural mechanisms involved in this social behaviour.

## Results

### Social buffering of fear in zebrafish–experiment I

**Figure 1:**
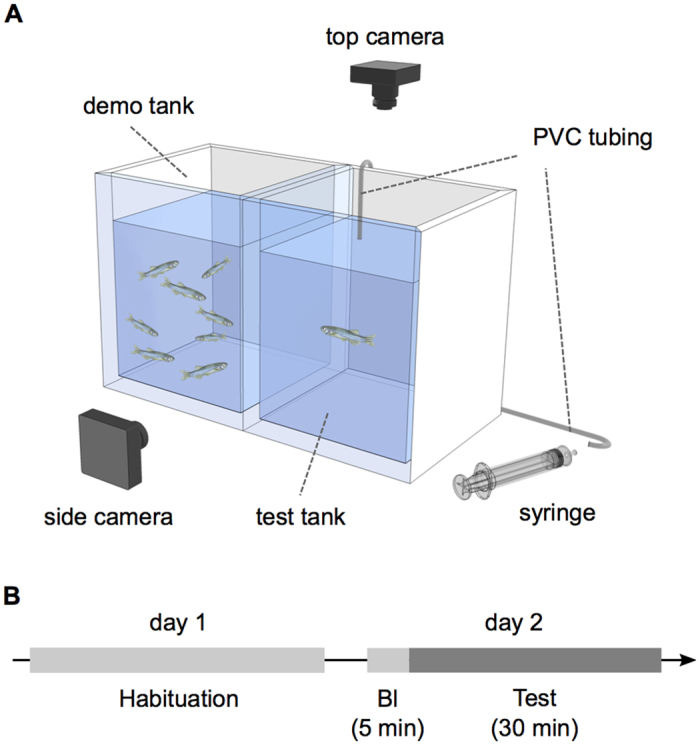
Behavioural paradigm for the study of SB in zebrafish: experimental setup and behavioural protocol schematics. (**A**) Schematic representation of the SB experimental setup. Test tank (focal fish) and demo tank (with shoal present or absent depending on the treatment) were side-by-side and physically separated. AS or water (depending on treatment) were administered through a PVC tubing with the help of a syringe. Behaviour was video recorded with side and top cameras. (**B**) Schematic representation of the behavioural protocol. On day 1 focal fish were left to habituate overnight to the experimental setup. On the following day (day 2), behavioural video recording was initiated with 5 min of baseline (Bl), followed by 30 min of test.

In this study we developed a behavioural paradigm to investigate SB in adult zebrafish when exposed to AS. To do so, focal fish (inside of the test tank) were exposed to AS in the absence or presence of shoal cues [olfactory (O) – i.e. shoal water added to the test tank; visual (V) – i.e. sight of shoal in the demonstrator (demo) tank; see Fig. 1 and Methods for details on the protocol] in order to test the hypothesis that the presence of conspecific cues decreases the fear response of the focal fish towards AS, comparatively to being alone. Focal fish behaviour was video recorded (see Fig. 1 and Supplementary Fig. 1A,B) both before (5 min baseline) and during exposure (30 min test) to AS, and stereotypic behavioural responses to AS (i.e. erratic movement and freezing^42^) were quantified using a custom-made software (xyz2b; see https://github.com/joseaccruz/xyz2b for detailed information and download). The xyz2b validation against a human observer for erratic movement and freezing quantifications showed the better performance of xyz2b in measuring freezing: *r* = 0.97, p < 0.001 (see Supplementary Fig. 2A,B and Supplementary Information for further details). Since freezing was the most frequent and consistent behaviour expressed throughout the 30 min test (Supplementary Fig. 2C; see Supplementary Information for further details), it was used as our behavioural measure of the AS-evoked fear response in all experiments.

**Figure 2:**
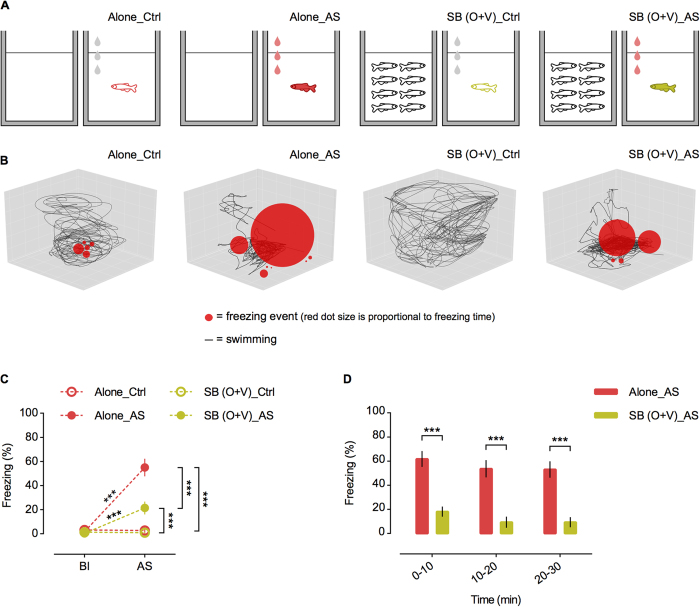
Experiment I. (**A**) Schematic representation of the behavioural treatments. From left to right: Alone_Ctrl–alone focal fish (red outline) administered with water; Alone_AS–alone focal fish (red filling) administered with AS; SB (O + V)_Ctrl–focal fish (green outline) administered with water and exposed simultaneously to shoal water and a shoal of 8 conspecifics, and SB (O + V)_AS–focal fish (green filling) administered with AS and exposed simultaneously to shoal water and a shoal of 8 conspecifics. Grey and red drops represent water and AS administration, respectively. (**B**) 3D plots representative of each behavioural treatment. Each 3D plot represents the first 5 min after AS onset for the focal fish closest to the mean in each treatment. n = 20 per treatment. Total freezing percentages presented (red circles) in each 3D plot are (from left to right): Alone_Ctrl–1.95%; Alone_AS–56.24%; SB (O + V)_Ctrl–0.00% and SB (O + V)_AS–23.32%. (**C**) Freezing % in baseline (Bl) vs. first 5 min after AS onset (AS). n = 20 per treatment. Mean ± SEM are shown. *p’ < 0.05; **p’ < 0.01 and ***p’ < 0.001. (**D**) Freezing % over the 30 min test in 10 min bins. n = 20 per treatment. Mean ± SEM are shown. *p’ < 0.05; **p’ < 0.01 and ***p’ < 0.001.

A two-factor 2 × 2 experimental design [social: alone, SB (O+V) (olfactory + visual shoal cues present); threat: exposed to AS, exposed to water] generated four treatments (Fig. 2A): Alone_Ctrl (alone control exposed to water); Alone_AS (alone exposed to AS); SB (O + V)_Ctrl (control with shoal cues and exposed to water); SB (O + V)_AS (with shoal cues and exposed to AS). Shoals of 8 conspecifics (4 females:4 males) were used since they represent medium-sized shoals observed in zebrafish floodplain habitats^43^,^44^ – the same holds for experiment II. Focal fish in the SB (O + V)_AS treatment presented significantly lower freezing behaviour than fish that were administered with the AS when alone (Alone_AS) during the whole 30 min exposure [repeated measures ANOVA with “time” as within-subject factor; time: F_(2,76)_ = 4.141, p = 0.020; treatment: F_(1,38)_ = 38.798, p < 0.001; treatment ∗ time: F_(2,76)_ = 0.006, p = 0.994; corrected p values (p’) after sequential Bonferroni correction for multiple comparisons (LSD post hoc) are reported – 0–10 min: p’ < 0.001; 10–20 min: p’ < 0.001; 20–30 min: p’ < 0.001; Fig. 2D]. Furthermore, there were no differences between the two control treatments Alone_Ctrl and SB (O + V)_Ctrl during the baseline (Bl), and the administration of water in the control treatments did not elicit freezing behaviour [repeated measures ANOVA with “time” as within-subject factor; time: F_(1.0, 76.0)_ = 68.418, p < 0.001; treatment: F_(3, 76)_ = 20.157, p < 0.001; treatment ∗ time: F_(3.0, 76.0)_ = 32.714, p < 0.001; *p’ < 0.05; **p’ < 0.01 and ***p’ < 0.001; Fig. 2C]. Importantly, the erratic movement was also significantly lower in the SB (O + V)_AS treatment comparatively to the Alone_AS treatment, but only in the first 5 minutes of the test, which further enhances the freezing parameter as the best behavioural readout to measure SB to AS-elicited responses in zebrafish (Supplementary Fig. 2D,E). These results show that the presence of conspecific cues reduces the fear response to AS, thus demonstrating the occurrence of social buffering of fear response in zebrafish (see Fig. 2D, Supplementary Movie 1 and Supplementary Movie 2).

### Effectiveness of visual and olfactory shoal cues at inducing social buffering–experiment II

**Figure 3:**
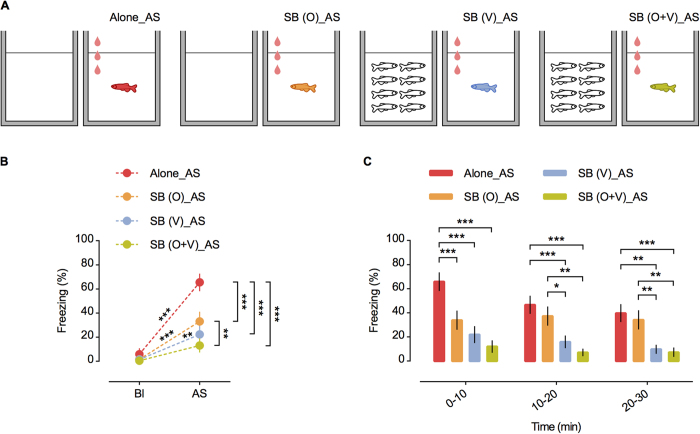
Experiment II. (**A**) Schematic representation of the behavioural treatments. From left to right: Alone_AS–alone focal fish (red filling) administered with AS; SB (O)_AS–focal fish (orange filling) administered with AS and exposed to shoal water of 8 conspecifics; SB (V)_AS–focal fish (blue filling) administered with AS and exposed to a shoal of 8 conspecifics and SB (O + V)_AS–focal fish (green filling) administered with AS and exposed simultaneously to shoal water and a shoal of 8 conspecifics. Red drops represent AS administration. (**B**) Freezing % in baseline (Bl) vs. first 5 min after AS onset (AS). n = 20 per treatment. Mean ± SEM are shown. *p’ < 0.05; **p’ < 0.01 and ***p’ < 0.001. (**C**) Freezing % over the 30 min test in 10 min bins. n = 20 per treatment. Mean ± SEM are shown. *p’ < 0.05; **p’ < 0.01 and ***p’ < 0.001.

SB can be mediated by social cues associated with different sensory modalities^7^,^8^,^9^. To our knowledge, research focused on disentangling the contribution of each sensory cue and its lasting effectiveness on the SB process has never been conducted before. Since in our first experiment the combined presentation of olfactory and visual shoal cues was effective in eliciting SB, we decided to investigate the specific role of each of these two sensory channels in this phenomenon. Although other sensory modalities may also modulate SB – tactile stimulation is known to reduce fear in zebrafish^45^ – we focused on the olfactory and visual channels, as they were easier to control experimentally. Thus, in a second experiment, we separately tested the effectiveness of olfactory (O) and visual (V) shoal cues on the fear response to the AS. For this purpose, zebrafish were exposed to AS in one of four treatments: Alone_AS; SB (O)_AS; SB (V)_AS; and SB (O + V)_AS (Fig. 3A; see Methods for further details). Whereas there were no differences in baseline freezing levels (i.e. before exposure to AS), either olfactory only, visual only, or both olfactory and visual shoal cues together were effective in reducing freezing behaviour after exposure to AS [repeated measures ANOVA with “time” as within-subject factor; time: F_(1, 76)_ = 74.352, p < 0.001; treatment: F_(3, 76)_ = 10.137, p < 0.001; treatment ∗ time: F_(3, 76)_ = 8.016, p < 0.001. *p’ < 0.05; **p’ < 0.01 and ***p’ < 0.001; Fig. 3B]. However, a more detailed analysis over time (30 min exposure) showed that although both olfactory and visual cues were equally effective in the first 10 min of the test, the visual cue was significantly more effective in decreasing the freezing response in the last 20 min [repeated measures ANOVA with “time” as within-subject factor; time: F_(1.6,120.625)_ = 11.723, p < 0.001; treatment: F_(3,76)_ = 10.284, p < 0.001; treatment ∗ time: F_(4.8,120.625)_ = 3.871, p = 0.003; corrected p values (p’) after sequential Bonferroni correction for multiple comparisons (LSD post hoc): O vs. V, 0–10 min: p’ = 0.194; O vs. V, 10–20 min: p’ = 0.021; O vs. V, 20–30 min: p’ = 0.009; Fig. 3C]. Moreover, in the last 20 min the visual cue was as efficient as the visual and olfactory cues combined, corroborating the higher effectiveness of the sight of shoal to the buffering phenomenon in long-lasting exposures to threat [corrected p values (p’) after sequential Bonferroni correction for multiple comparisons (LSD post hoc): V vs. O + V, 0–10 min: p’ = 0.281; V vs. O + V, 10–20 min: p’ = 0.338; V vs. O + V, 20–30 min: p’ = 0.788; O vs. O + V, 0–10 min: p’ = 0.019; O vs. O + V, 10–20 min: p’ = 0.001; O vs. O + V, 20–30 min: p’ = 0.004; Fig. 3C].

### Smaller shoals are equally effective in promoting social buffering–experiment III

**Figure 4:**
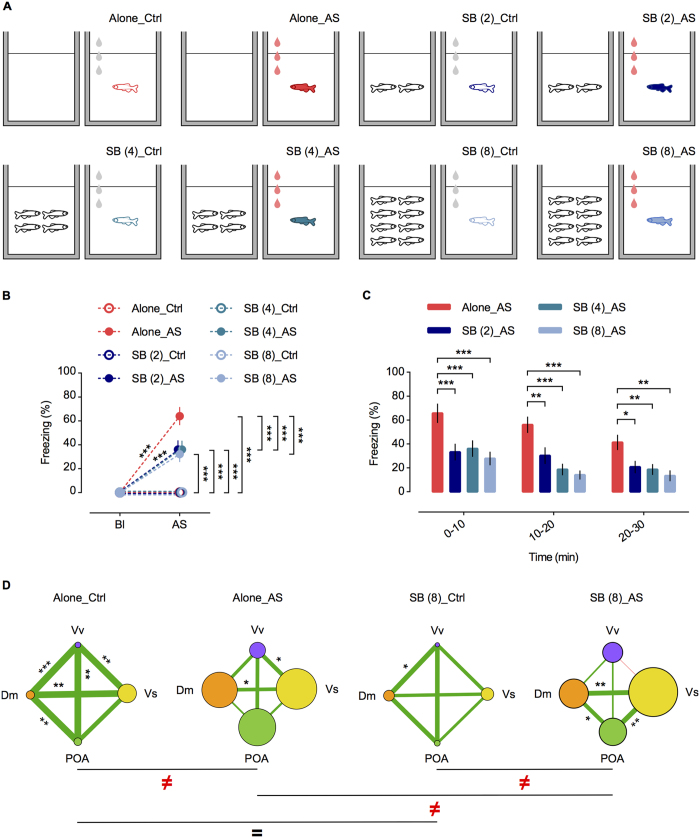
Experiment III. (**A**) Schematic representation of the behavioural treatments. From left to right (top panel): Alone_Ctrl–alone focal fish (red outline) administered with water; Alone_AS–alone focal fish (red filling) administered with AS; SB (2)_Ctrl–focal fish (dark blue outline) administered with water and exposed to a shoal of 2 conspecifics; SB (2)_AS–focal fish (dark blue filling) administered with AS and exposed to a shoal of 2 conspecifics. From left to right (bottom panel): SB (4)_Ctrl–focal fish (sea blue outline) administered with water and exposed to a shoal of 4 conspecifics; SB (4)_AS–focal fish (sea blue filling) administered with AS and exposed to a shoal of 4 conspecifics; SB (8)_Ctrl–focal fish (light blue outline) administered with water and exposed to a shoal of 8 conspecifics; SB (8)_AS–focal fish (light blue filling) administered with AS and exposed to a shoal of 8 conspecifics. Grey and red drops represent water and AS administration, respectively. (**B**) Freezing % in baseline (Bl) vs. first 5 min after AS onset (AS). n = 20 per treatment. Mean ± SEM are shown. *p’ < 0.05; **p’ < 0.01 and ***p’ < 0.001. (**C**) Freezing % over the 30 min test in 10 min bins. n = 20 per treatment. Mean ± SEM are shown. *p’ < 0.05; **p’ < 0.01 and ***p’ < 0.001. (**D**) Brain networks as measured by *c-fos* mRNA expression for each treatment. Circle diameters represent the mean *c-fos* expression for each brain nuclei. Distinct (**≠**) and similar (=) co-activation patterns of *c-fos* mRNA expression between treatments are indicated. Lines linking brain nucleus represent the co-activation between them, as revealed by Pearson’s correlation coefficients (*r*), with line thicknesses proportional to *r* value and positive/negative correlations indicated by line colour (green and red, respectively); asterisks indicate significant correlations: *p < 0.05; **p < 0.01 and ***p < 0.001.

Given the greater efficacy of the sight of the shoal to the buffering behaviour, on a third experiment we tested if shoal size modulates the effectiveness of the visual cue in reducing the fear response. Shoal size was manipulated (mixed-sex shoals of 2, 4 and 8 conspecifics) such that 8 experimental treatments were generated: Alone_Ctrl; Alone_AS; SB (2)_Ctrl; SB (2)_AS; SB (4)_Ctrl; SB (4)_AS; SB (8)_Ctrl; SB (8)_AS – where controls were exposed to water (Fig. 4A; see Methods for further details). We verified that conspecific number did not influence freezing responses during the entire duration of the test (Fig. 4C), and that a shoal of 2 conspecifics was enough to significantly decrease freezing behaviour in response to the AS [repeated measures ANOVA with “time” as within-subject factor; time: F_(1.6,122.349)_ = 19.234, p < 0.001; treatment: F_(3,76)_ = 9.822, p < 0.001; treatment ∗ time: F_(4.8,122.349)_ = 1.232, p = 0.299; corrected p values (p’) after sequential Bonferroni correction for multiple comparisons (LSD post hoc): alone vs. shoal 2, 0–10 min: p’ < 0.001; alone vs. shoal 2, 10–20 min: p’ = 0.003; alone vs. shoal 2, 20–30 min: p’ = 0.016; Fig. 4C]. Again, we found no differences between control treatments and the administration of water did not elicit freezing behaviour [repeated measures ANOVA with “time” as within-subject factor; time: F_(1.0,152.0)_ = 131.542, p < 0.001; treatment: F_(7,152)_ = 22.454, p < 0.001; treatment * time: F_(7.0,152.0)_ = 22.358, p < 0.001. *p’ < 0.05; **p’ < 0.01 and ***p’ < 0.001; Fig. 4B].

### Neuromolecular mechanisms underlying social buffering

*c-fos* is a transient marker of neuronal activity^46^,^47^ and its expression has been used to characterize brain activation in response to behavioural manipulations^23^,^48^,^49^. Thus, continuing our third experiment, we used *c-fos* expression to characterize the pattern of neuronal activation during SB across a set of brain nuclei that are putative homologues to those in mammals^25^,^26^,^27^,^28^,^29^ involved in SB^4^,^50^, fear^32^,^33^,^34^,^35^, anxiety^35^,^36^,^37^ and social behaviour regulation^38^,^39^,^40^,^41^, namely: the medial part of the dorsal telencephalon (Dm, homologue of the mammalian pallial amygdala), the supracommissural nucleus of the ventral telencephalon (Vs, homologue of the mammalian subpallial amygdala), the ventral nucleus of the ventral telencephalon (Vv, homologue of the mammalians nucleus accumbens and septum), and the preoptic area (POA, homologue of the mammalian preoptic area/paraventricular nucleus)^25^,^26^,^27^,^28^,^29^. Although the habenula (Hb) has been implicated in fear responses in zebrafish^18^,^19^, the microdissection technique used in this study did not allow us to accurately sample this brain region. Thus, we microdissected (see Supplementary Fig. 3) the above-mentioned brain nuclei (except Hb) in fish from experiment III (treatments Alone and SB (8) were used, see Fig. 4A), and the expression levels of *c-fos* on each brain nucleus were measured using quantitative RT-PCR (see Methods for further information). A shoal size of 8 was selected for characterizing the brain activation pattern during SB, since experiment III revealed that all shoal sizes equally modulate buffering and this group size is the closest to the average shoal size observed in zebrafish common floodplain habitats^43^,^44^,^51^. Differences in co-activation patterns between treatments were assessed by testing the association between the correlation matrices for *c-fos* expression across brain nuclei for each treatment, using the quadratic assignment procedure (QAP)^52^. Co-activation patterns among areas were also characterized using cohesion and centrality network measures (see Methods for more details). There was a significant increase in activity in almost all brain regions measured (except for Vv when shoal was present) when the fish were exposed to the AS, either alone or in the presence of conspecifics, but there were no significant differences in the activation of any of the regions between fish exposed to AS alone or in the presence of the shoal [one-way ANOVA; F_(12,90.25)_ = 2.929, p < 0.01; corrected p values (p’) after sequential Bonferroni correction for multiple comparisons (LSD post hoc): Dm: p’ = 0.802; Vv: p’ = 0.725; Vs: p’ = 0.361; POA: p’ = 0.593; Supplementary Fig. 4]. In contrast, the co-activation patterns across brain nuclei were specific for each treatment except for the two control treatments [as indicated by QAP correlations – Alone_AS vs. SB (8)_AS: *r* = −0.663, p = 0.086; SB (8)_Ctrl vs. SB (8)_AS: *r* = −0.437, p = 0.256; Alone_Ctrl vs. Alone_AS: *r* = 0.480, p = 0.248; Alone_Ctrl vs. SB (8)_Ctrl: *r* = 0.763, p = 0.045; see Fig. 4D and Methods for details regarding the QAP analysis]. Thus, SB coincides with a specific pattern of brain co-activation, characterized by significant correlations between Dm-Vs-POA. Moreover, the SB (8)_AS treatment (i.e. shoal of 8 exposed to AS) was the only one where network measures were significantly different, with Vv presenting lower centrality than the other nuclei, therefore suggesting that Vv is decoupled from the other brain regions in the response to AS when conspecifics are present (see Supplementary Table 2 for more information). We speculate that this less central role of Vv in SB is related to the central involvement of this area in the regulation of anxiety-like and social rewarding behaviours^38^,^39^,^40^,^41^, while SB seems to be mainly associated with homologous brain regions of those involved in fear and stress responses in mammals^2^,^6^,^8^,^9^.

## Discussion

Individuals recover better from aversive events when conspecifics are present, a phenomenon that is called social buffering (SB)^2^,^4^,^53^. Although there is some literature addressing the buffering behaviour in mammals^4^,^6^,^8^, research in other vertebrate taxa is very limited^3^ and comparative studies are essential to understand the evolution of SB and its mechanisms among vertebrates. For this reason, we studied SB of fear in zebrafish, a vertebrate social species with an established alarm fear-like response^12^,^14^,^15^,^43^, that responds to both olfactory and visual cues of fear in conspecifics^16^,^17^, and currently provides a wide set of neurogenetic tools for the investigation of neural mechanisms underlying behaviour^18^,^19^,^20^,^23^,^24^. Our results showed that zebrafish decreased their freezing behaviour towards AS in the presence of conspecifics’ cues (olfactory + visual). Our data provides robust evidence on the SB phenomenon in zebrafish and suggests that SB in vertebrates may share a common evolutionary origin. A previous study using zebrafish *gr*^*s357*^ mutants (a mutation that induces the disruption of the feedback on the stress response due to the complete abolition of all transcriptional activity of glucocorticoid receptor – GR) showed that the presence of conspecifics reduces the freezing response towards social isolation^54^. However the buffering effect was not verified in the heterozygous fish and no effect on the wild phenotype was reported^54^, suggesting that the obtained buffering response was probably associated with the disruption of GR genomic activity.

After demonstrating SB in zebrafish, we investigated the efficacy of the different sensory modalities in the buffering behaviour. It is already known that conspecific sensory cues also contribute to SB by aiding in the dampening of fear and stress responses in other species^7^,^8^,^9^. However, the contribution of different sensory modalities to the same buffering episode and the effectiveness of different sensory cues in short and long-term exposures to a threat had never been addressed. In our study, we observed that even though both olfactory and visual cues equally contribute to the buffering phenomenon in a short-term exposure to AS, a greater efficacy of the visual cue was observed in a long-term exposure to the aversive stimulus. Thus, our results suggest that the long-term reliability of shoal cues signalling safety depends on sensory modality, and this may be due to the refresh rate of information typical of each sensory channel. Since it was previously shown that the value of the social information available is dependent on its reliability^55^, we speculate that in an initial phase of exposure to the AS both visual and olfactory shoal cues are equally reliable, since both indicate the presence of peers. However, as time goes by, available visual information from the shoal is constantly updated (that is, the shoal is a dynamic visual cue resembling safety, as relaxed shoal mates indicate a harmless environment), contrary to the case of olfactory cues where information will remain unaltered in the water, therefore preventing updates in the olfactory channel. Hence, we suggest that after an initial phase, visual cues are expected to be more reliable than olfactory ones in signalling a secure environment, contributing to zebrafish’ survival in long-lasting exposures to threat.

Furthermore, it is known from the literature that species form larger groups in response to threats^56^ and that bigger group sizes modulate responses to aversive events^30^,^31^. Thus, we hypothesized that the number of conspecifics in a shoal could potentially influence the effectiveness of the visual cue in promoting SB, as a greater number of animals are conveying the same information of safety. Therefore, in a third experiment, we tested the role of shoal size modulation in the buffering behaviour, since larger groups may be more conspicuous and reliable. In this experiment, zebrafish were exposed to AS in the presence of different mixed-sex shoal sizes (2, 4 and 8 fish). We observed that all shoals equally contributed to the buffering effect, with smaller shoals of two conspecifics proving to be enough to decrease the fear responses elicited by AS, thus showing that SB is independent of shoal size. In our experiment, the larger shoal size tested consisted of 8 conspecifics. Since larger shoals of up to 30 individuals have been observed in zebrafish floodplain habitats^44^, we cannot rule out the possibility that much larger shoals (e.g. 20, 30) may be even more efficient in buffering fear responses, since fish in larger groups (i.e. 20, 50) are known to recover faster from a threatening event than fish in smaller groups (i.e. 10)^30^. Accordingly, studies addressing the effect of greater shoal sizes (e.g. 30) in the buffering phenomenon should be conducted in the future.

By determining the efficacy of different sensory modalities and the effect of shoal size in the SB phenomenon, we established a robust sensory stimulus that greatly contributes to the SB phenomenon in zebrafish and enables the accurate dissection of the neural mechanisms underlying this behaviour. Since it has been recently shown that behavioural states in zebrafish are better associated with patterns of co-activation of relevant brain nuclei than with localized levels of activation of specific brain regions^23^, we have analyzed the effects of SB both on the activation levels of each of the brain nucleus per se and on the pattern of co-activation across nuclei. Although there were no differences in the activation of any of the regions between fish exposed alone to AS or in the presence of the shoal, SB elicited a specific co-activation pattern, characterized by a significant functional connectivity between Dm-Vs-POA–brain nuclei known to be involved in buffering responses in mammals^2^,^4^,^6^,^8^,^9^, rather than by a localized increase in activity in a single brain nucleus. A recent study has also documented similar findings in a different behavioural context (aggression paradigm^23^), possibly indicating that different social behaviours reflect an overall profile of activation across different brain nuclei instead of single node activation. Additionally, these changes in co-activation patterns across a set of zebrafish brain regions that are putative homologues of brain nuclei in mammals, and are important in activating a response to psychogenic threats^35^,^57^,^58^,^59^, suggest a substantially evolutionary conserved mechanism underlying SB in two distinct vertebrate lineages.

As the SB phenomenon seems to be eliciting the co-activation of several brain areas rather than the activation of localized brain regions, brain imaging and optogenetic techniques^19^,^60^,^61^,^62^,^63^ would greatly benefit the understanding of this social behaviour at the circuit level. However, these techniques are optimized for zebrafish larval stages and most studies in zebrafish addressing social behaviours (including the present one) have been performed in adults^22^,^23^,^24^,^64^,^65^. Fortunately, recent studies have shown a strong visual preference for conspecifics in 3 weeks old larvae^66^,^67^, suggesting that social behaviours appear earlier in zebrafish development. Thus, some recent studies have started to use imaging techniques in 21–28 days^60^ and 3–5 weeks zebrafish larvae^68^. Accordingly, it is plausible to suggest that the study of SB in zebrafish younger stages would greatly benefit the investigation of the neural circuits at the basis of this phenomenon. Our results set the stage for such exploration, as we provide evidence on the behavioural, sensory and neural mechanisms associated with the buffering phenomenon, establishing zebrafish as an ideal genetically tractable vertebrate model for the study of SB’s neural basis.

In conclusion, we found that zebrafish decreased their fear responses in the presence of conspecific cues, and that sight of conspecifics was more effective than their odour in promoting SB in a persistent threat scenario. Moreover, we showed that SB was independent of conspecific number and is paralleled by a specific pattern of co-activation of homologue brain regions to those involved in the same phenomenon in mammals, suggesting not only that SB in vertebrates may share a common evolutionary origin, but also that social support during a threatening event seems to be a conserved process across species.

## Methods

### Fish and Housing

All subjects used were 6–9 months old male wild-type (TU) zebrafish (*Danio rerio*) bred and held at Instituto Gulbenkian de Ciência (IGC, Oeiras, Portugal). Fish were kept at 28 °C, 750 μS, 7.0 pH in a 14 L:10D photoperiod and fed twice a day (except on the day of the experiments) with freshly hatched *Artemia salina* in the morning and commercial food flakes in the afternoon (see Supplementary Information for more details on fish and housing procedures). All experiments were performed in accordance with the relevant guidelines and regulations, reviewed by the Instituto Gulbenkian de Ciência Ethics Committee, and approved by the competent Portuguese authority (Direcção Geral de Alimentação e Veterinária, permit 008955).

### Experiments

The experimental setup (see Fig. 1) consisted of a test and a demo tank. Two cameras were placed on the side and top of each test tank to acquire side and top view recordings of the test and demo tanks simultaneously. Test (containing a focal fish) and demo tanks (empty tank or shoal present, depending on the treatment) were placed side-by-side but physically separated (see Fig. 1), and focal fish (but not shoals) were exposed to AS (see Supplementary Information for further details on AS preparation). In all experiments, focal fish were left to habituate overnight in the test tank in visual contact with the demo tank) (day 1), and the behaviour test was conducted on the following day (day 2). Shoal members (demo tank) were always siblings and tank mates of the focal fish to avoid possible confounding effects of familiarity^2^ (the same holds for experiments II and III). We defined familiarity as being kin and tank mates (both in raising and stock tanks. In all experiments, 5 min (baseline period) after video recording was initiated, 0.754 mL of filtered water (control treatments) or 0.754 mL of AS (treatments with AS) was delivered to the test tank (Fig. 1). The test lasted for 30 min, after which each focal fish were immediately euthanized with an overdose of tricaine solution (MS222, Pharmaq; 500–1000 mg/L). Test and demo tanks were sprayed with 70% ethanol and rinsed with filtered water between treatments, to eliminate hormone and odour residues. All test trials were conducted between 10:30 a.m. and 07:30 p.m., and the different experimental groups were intermixed throughout the day to account for possible diurnal variations in behaviour (see Supplementary Information for more details on the experimental procedures).

### Video tracking and behavioural analysis

The x, y, z coordinates of focal fish were extracted for each frame using a commercial video tracking software (EthoVision^®^ XT 8.0, Noldus Inc. the Netherlands) and were then fed into the xyz2b software. To test the performance of xyz2b detecting erratic movement and freezing behaviours, the code was validated against a human observer using a multi-event recorder (Observer^®^ XT 7.0, Noldus; for more details on video tracking and behavioural analysis see Supplementary Information).

### Brain microdissection protocol

Only fish from the treatments Alone_Ctrl, Alone_AS, SB (8)_Ctrl and SB (8)_AS of experiment III were subjected to the brain microdissection protocol. Immediately after the experiment, fish were euthanized with an overdose of tricaine solution (MS222, Pharmaq; 500–1000 mg/L) followed by rapid decapitation. Zebrafish heads were embedded in mounting media (OCT Compound, Tissue Tek, Sakura), rapidly frozen on dry ice, and stored at −80 °C until further processing. For brain microdissection, zebrafish heads were retrieved from −80 °C and sliced on a cryostat (Leica CM 3050S) in serial 150 μm-thick coronal sections that were collected onto regular glass slides previously cleaned with 70% ethanol. Once all sections of interest were sampled, they were microdissected under a stereoscopic microscope (Nikon SMZ745) on top of a cold plate. Brain nuclei were identified, classified as in ref. 69, and harvested with a modified 27G needle. The harvested tissue was immediately collected into QIAzol Lysis Reagent (Qiagen) and stored at −80 °C until gene expression analysis procedure (see Supplementary Information for further details on the brain microdissection protocol).

### Gene expression analysis

RNA extraction was performed with RNeasy^®^ Lipid Tissue Mini Kit using the manufacturer protocol with minor modifications. iScript™ cDNA Synthesis Kit (Bio-Rad) was used to synthesize the DNA in accordance with manufacturer’s instructions. qPCR (quantitative polymerase chain reaction) was performed to determine mRNA expression levels of *c-fos* (immediate early gene) and *18s rRNA* (reference gene). For further information on gene expression analysis see Supplementary Information, particularly the Supplementary Table 1 for primer sequences.

### Statistical Analysis

Behavioural and relative gene expression statistical analysis were performed on the statistical software packages STATISTICA v. 10 (StatSoft, Inc.) and SPSS^®^ Statistics v. 21 (IBM). Repeated measures ANOVAs and a one-way ANOVA were used to determine differences between behavioural treatments and relative gene expression, respectively, followed by LSD posthoc. All pairwise comparisons extracted from the LSD posthoc matrix were corrected for multiple comparisons using the sequential Bonferroni correction. Brain co-activation of the different treatments was assessed as in a previous study^23^. The occurrence of different patterns of brain co-activation associated with different behavioural treatments was determined by testing the association between any two Pearson correlation matrices using the quadratic assignment procedure (QAP) correlation test^23^. Correlation matrices were considered different when QAP p-value was higher than 0.05^52^. Finally, cohesion and centrality network measures (density and eigenvector centrality, respectively) were used to structurally characterize the brain network underlying each treatment^70^. Network statistical analysis was performed using UCINET 6^70^. Brain nuclei co-activation network figures (Fig. 4D) were produced using a custom-made python code (see Supplementary Information for detailed information on statistical analysis).

## Additional Information

**How to cite this article**: Faustino, A. I. *et al*. Mechanisms of social buffering of fear in zebrafish. *Sci. Rep.* **7**, 44329; doi: 10.1038/srep44329 (2017).

**Publisher's note:** Springer Nature remains neutral with regard to jurisdictional claims in published maps and institutional affiliations.

## Supplementary Material

Supplementary Information

Supplementary Video 1

Supplementary Video 2
