# Supplementary material for: Mechanisms of social buffering of fear in zebrafish
Source: Sci Rep. 2017 Mar 31;7:44329. doi: 10.1038/srep44329 (PMC5374490; doi:10.1038/srep44329)
Supplement: Supplementary Information [file srep44329-s1.pdf]

Supplementary information

Mechanisms of social buffering of fear in zebrafish

Ana I. Faustino, André Tacão-Monteiro and Rui F. Oliveira

Supplementary figures

Supplementary Figure 1

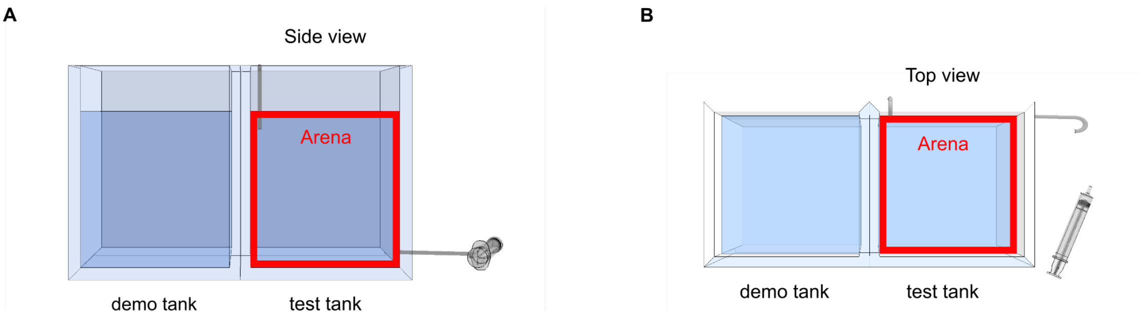

Figure 1. Side and top view of 2D arenas. A 10.5 × 11 cm 2D arena was defined for tracking using a side view (**A**); and a 10.5 × 10.5 cm 2D arena was defined for the top view (**B**). Both arenas excluded glass walls thickness and all fish were tracked at 25 frames per second. The video tracking software EthoVision was used to determine and extract fish coordinates for posterior behavioural data analysis using xyz2b.

Supplementary Figure 2

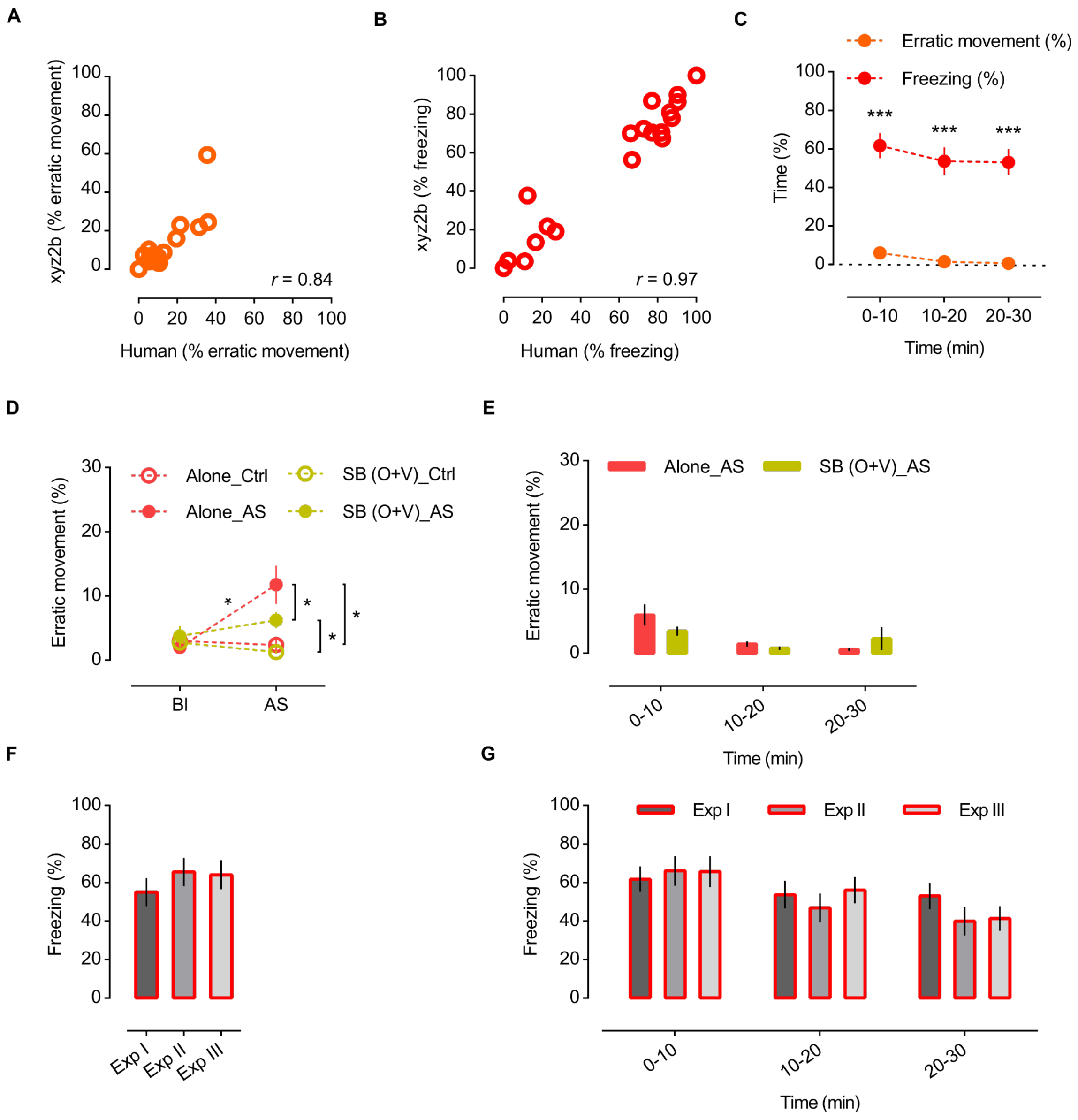

Figure 2. Freezing is the most accurate behavioural parameter to quantify the SB phenomenon and freezing % is stable across experiments independently of the AS set used. (**A-B**) Validation of xyz2b against a human observer. To test the performance of xyz2b detecting erratic movement and freezing behaviours, the code was validated against a human observer using a multi-event recorder (Observer® XT 7.0, Noldus) for 20 videos (first 5 min) of the treatment Alone_AS of experiment I. xyz2b and human quantifications of (**A**) erratic movement and (**B**) freezing were positively correlated ($r = 0.84$, $p < 0.001$ and $r = 0.97$, $p < 0.001$, respectively), which validates the use of xyz2b to measure these behaviours. (**C**) Erratic movement vs. Freezing. In order to assess the parameter that better quantifies zebrafish response to AS, erratic movement and freezing behaviours were compared during the 30 min test (10 min bins). Freezing was the most frequent and consistent parameter over time, with higher percentages of freezing behaviour in all the 10 min bins of the entire 30 min test ($p' < 0.001$). Repeated measures ANOVA showed differences for behaviour $F_{(1, 38)} = 77.189$; $p < 0.001$ and time $F_{(2, 76)} = 3.713$; $p < 0.05$. No differences were found for the interaction between behaviour and time $F_{(2, 76)} = 0.246$; $p = 0.783$. * $p' < 0.05$; ** $p' < 0.01$ and *** $p' < 0.001$. Analysis performed on 20 videos from the treatment Alone_AS of the experiment I. (**D-E**) Freezing is the best parameter to assess SB in a persistent threat scenario. (**D**) Acute response

to AS - first 5 min. (**E**) Sustained response to AS - 30 min. (**D-E**) To further confirm freezing as the best behavioural readout to measure SB we quantified the erratic movement behaviour in the acute and long-term response to AS in the presence [SB (O+V)_AS] or absence (Alone_AS) of conspecific cues (experiment **I**). The results demonstrate that the erratic movement was significantly lower in the SB [SB (O+V)_AS] treatment comparatively to the alone treatment (Alone_AS), but only in the first 5 minutes of the test. * p' < 0.05; ** p' < 0.01 and *** p' < 0.001. Repeated measures ANOVA for the acute response - first 5 min: time: $F_{(1.0,\ 76.0)} = 7.029$; p = 0.01; treatment: $F_{(3,\ 76)} = 4.86$; p = 0.004; treatment*time: $F_{(3.0,\ 76.0)} = 7.163$; p < 0.001. Repeated measures ANOVA for the sustained response - 30 min, analyzed in 10 min bins: time: $F_{(1.63,\ 62.10)} = 7.644$; p = 0.002; treatment: $F_{(1,\ 38)} = 0.310$; p = 0.581; treatment*time: $F_{(1.63,\ 62.10)} = 2.095$; p = 0.140. This result further enhances freezing as the best behavioural readout to measure SB to AS-evoked responses in zebrafish, particularly in longer exposures to AS as was the case of our behavioural experiment. (**F-G**) AS efficiency across experiments. (**F**) Neither the acute [first 5 min: $F_{(2,\ 57)} = 0.587$; p = 0.560] nor (**G**) the sustained [30 min, analyzed in 10 min bins: $F_{(2,\ 57)} = 0.182$; p = 0.834] freezing response to the AS varied across experiments. Alone_AS treatment data was used for this analysis. These results showed that when the skin extract preparation that conveys the AS is prepared controlling for sex-ratio, age and a total number of donor fish, there is no significant variation in AS efficiency.

Supplementary Figure 3

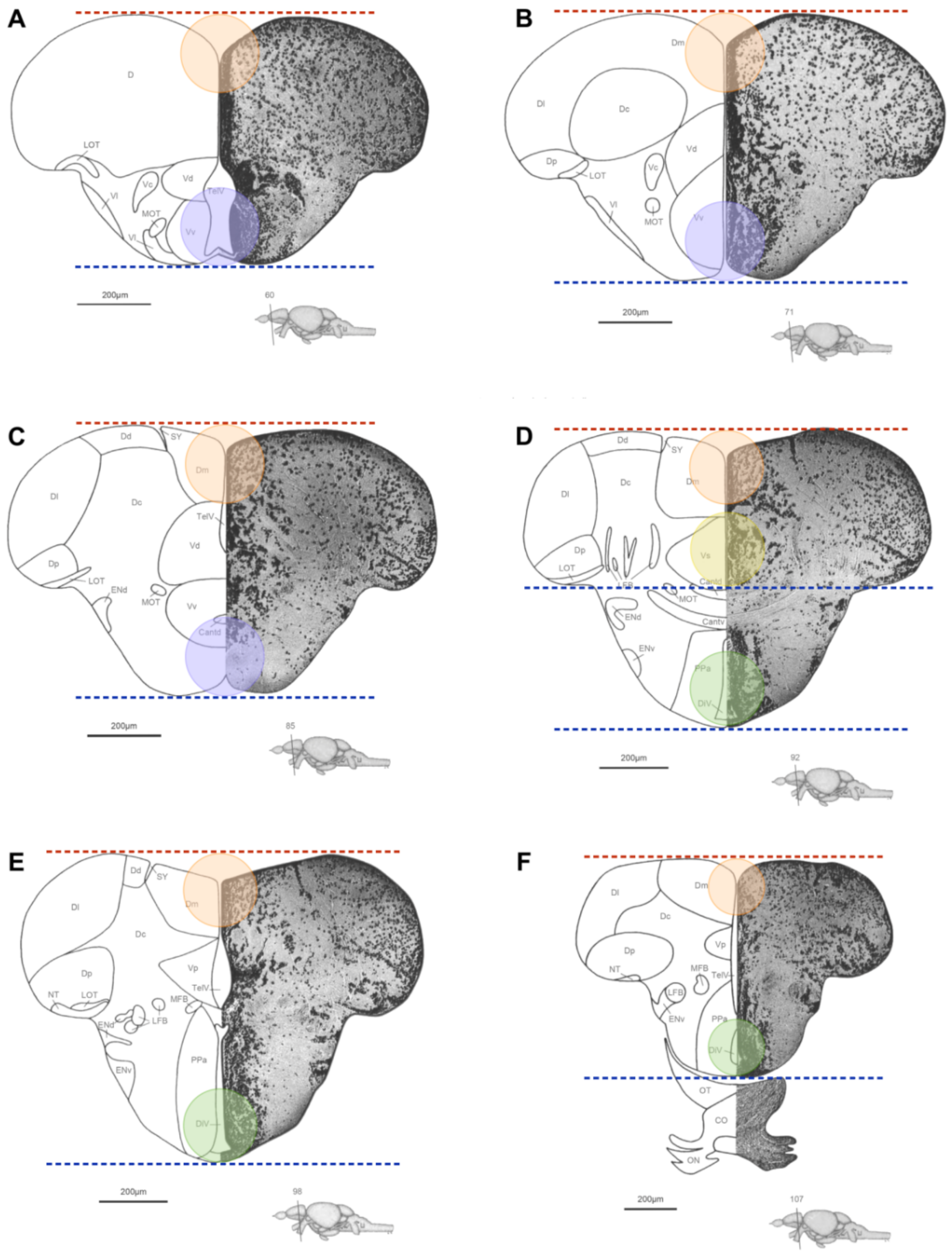

Figure 3. Microdissection of regions of interest in the zebrafish brain. Figure adapted from Wullimann, M. F., Rupp, B. & Reichert, H. *Neuroanatomy of the Zebrafish Brain: A Topological Atlas*. (Birkhäuser Basel, 1996). doi:10.1007/978-3-0348-8979-7[1], with permission of Springer Nature. This figure is not covered by the CC BY licence. All rights reserved, used with permission. (**A-F**) Coronal sections from the zebrafish brain are represented. The right hemisphere shows tissue sections stained with Nissl-stain cresyl violet and the left hemisphere a schematic representation of the sections with each nucleus specified and delimitated. In the bottom right of each coronal section, a lateral view of the brain with the location of the respective section (numbers indicate the amount of 10 µm sequential slices made to reach the observed image) is presented. Circles indicate the sampling points for the target brain nuclei in the various sections, and their diameter is scaled for the inner diameter of the microdissection

needle; orange circles represent sampling points for the medial zone of the dorsal telencephalic area (Dm); purple circles represent sampling points for the ventral nucleus of the ventral telencephalic area (Vv); yellow circles represent sampling points for the supracommissural nucleus of the ventral telencephalic area (Vs) and green circles represent sampling points for the preoptic area (POA). Red and blue dashed lines indicate top and bottom reference limits for samples microdissection, respectively. It is important to note that in this study, microdissections were performed in sections of 150 µm, therefore the coronal sections represented (10 µm) are only a reference and each sampling point may comprise tissue from more than one of the sections presented.

Supplementary Figure 4

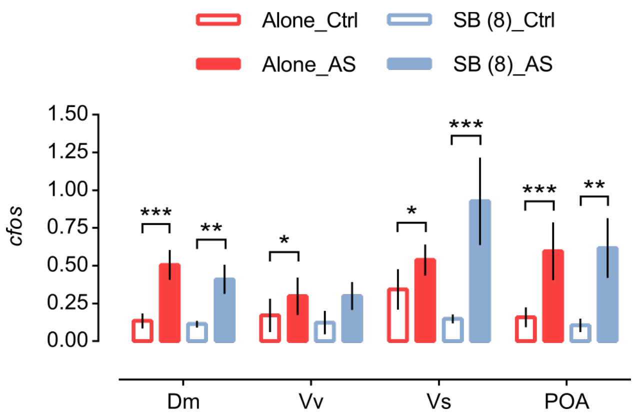

Figure 4. *c-fos* gene expression in different brain nuclei. (Dm, medial zone of the dorsal telencephalic area; Vv, ventral nucleus of the ventral telencephalic area; Vs: supracommissural nucleus of the ventral telencephalic area; POA, preoptic area) for different behavioural treatments [Alone_Ctrl, Alone_AS, SB (8)_Ctrl and SB (8)_AS]. *c-fos* gene expression was normalized to *18S.* Bars represent mean ± SEM. One-way ANOVA analysis revealed an overall effect for *c-fos*: $F_{(12, 90.25)} = 2.929$, $p < 0.01$. However there was no evidence of SB in *c-fos* gene expression in any of the brain nuclei considered (Dm: p' = 0.802; Vv: p' = 0.725; Vs: p' = 0.361; POA: p' = 0.593). Interestingly, AS significantly elicited *c-fos* expression in both Alone_AS and SB (8)_AS treatments across all brain nuclei (except for Vv when shoal was present). * p' < 0.05; ** p' < 0.01 and *** p' < 0.001.

Supplementary tables

Supplementary Table 1: Primer sequences, amplicons length and annealing parameters for the genes used in qPCR.

Gene	Accession Number	Primer Sequence (5'→3')	Amplicon length (bp)	Ta (°C)	Time of annealing (s)	Source
18s	NM_173234.1	For – GCACATCCTTCGTGTCCTCAA<br>Rev – ACCCTCTCAACCTCATCCTCA	171	61	30	Current paper
c-fos	NM_205569.1	For – CCGATACACTGCAAGCTGAA<br>Rev – CGGCGAGGATGAACTCTAAC	111	58	60	2

For – primer forward; Rev – primer reverse; Ta – annealing temperature

Supplementary Table 2: Characterization of the co-activation (i.e. *c-fos* mRNA expression) patterns of the four brain regions sampled. Eigenvector centrality (eigenvalue) and density were used as centrality and cohesion measures, respectively.

	Brain Nucleus	Treatment			
		Alone_Ctrl	Alone_AS	SB (8)_Ctrl	SB (8)_AS
**eigenvalue**					
	Dm	0.524	0.472	0.529	0.557
	Vv	0.524	0.560	0.536	0.196
	Vs	0.473	0.529	0.424	0.575
	POA	0.476	0.429	0.503	0.566
**density**					
		0.813	0.506	0.501	0.448

Alone_Ctrl: alone focal fish administered with water; Alone_AS: alone focal fish administered with AS; SB (8)_Ctrl: focal fish administered with water and exposed to a shoal of 8 conspecifics; SB (8)_AS: focal fish administered with AS and exposed to a shoal of 8 conspecifics; Dm: medial zone of the dorsal telencephalic area; Vv: ventral nucleus of the ventral telencephalic area; Vs: supracommissural nucleus of the ventral telencephalic area; POA: preoptic area.

Supplementary methods

Fish and Housing. All fish were raised in groups of 35 individuals (29 males, 6 females) in 3.5 L tanks (raising tanks), in a recirculating system (ZebTec, 93 Tecniplast). Holding-water was monitored for nitrites (<0.2 ppm), nitrates (<50 ppm) and ammonia (0.01-0.1 ppm). At least one week before experiments, fish were transferred to the behavioural room and housed in 6 L tanks (30 × 15 × 17 cm) under the same environmental conditions, in groups of 30 individuals (24 males, 6 females). This fish density was established to fulfil a 5 fish per litre criteria, thus decreasing agonistic interactions and lowering cortisol levels of fish in the stock tanks[3]. By implementing this density, space for fish to establish territories[4] was diminished; therefore

promoting environmental conditions for focal individuals to have less social status discrepancies (control for the influence of hierarchical background in the response to AS). The 6 L tanks were environmentally enriched with a 1 cm layer of crushed shells substrate.

Experiments

Experimental setup. The experimental setup (see Fig. 1 of the main text) consisted of test and demo tanks pairs (12 × 12 × 15 each) placed side-by-side. This setup was replicated eight times as to perform all the treatments on the same day, in a randomized manner. The observation side of each test tank faced the demo tank and both test and demo tanks had a white opaque bottom and two white opaque walls (observation and the camera sides were kept transparent) to avoid visual contact with nearby tanks and interference of undesired visual cues from either the experimenter or the behavioural room. A 60 cm long, flexible and transparent PVC tubing (0.8 mm internal diameter; 2.4 mm external diameter) was introduced into each test tank (1 cm underwater) to enable the administration of the AS. At the PVC tubing's farthest extremity (from the test tank), a 2-10 µL transparent pipette tip was attached to facilitate AS administration. All tanks were filled with 1.3 L of water. Two B&W mini surveillance cameras (Henelec 300B), connected to a laptop (Samsung NP350V5C) through a USB 2.0 video capture device (ezcap), were placed on the side and on top of each test tank to acquire side and top view recordings of the test and demo tanks simultaneously. Video synchrony between the side and top cameras was possible using Bonsai (https://bitbucket.org/horizongir/bonsai), an open source visual programming framework for data stream processing. A black curtain partially covered the top part of the setup to avoid light incidence and no person was allowed inside the behavioural room during experiments (apart from the experimenter) to maintain quiet experimental conditions.

Experiment I. A total of 80 focal naïve male zebrafish were used (20 per treatment). Each focal fish was submitted to a single test corresponding to one of four possible treatments (see main Fig. 2A): alone focal fish administered with water (Alone_Ctrl); alone focal fish administered with AS (Alone_AS); focal fish administered with water and exposed simultaneously to shoal water and a shoal of 8 conspecifics [SB (O+V)_Ctrl]; and focal fish administered with AS and exposed simultaneously to shoal water and a shoal of 8 conspecifics [SB (O+V)_AS]. With our behavioural setup, we were able to test eight focal fish per day (two fish per treatment). The control treatments allowed us to verify possible fear inducing responses introduced by the administration procedure in the experimental protocol. On the afternoon of the day before the test, fish were randomly removed from their stock tanks and isolated in each test tank overnight. Per session, the order of the treatments attributed to each tank was done in a randomized fashion. To prepare the Alone_Ctrl and the Alone_AS treatments, 1.3 L of filtered water were placed both in the test and demo tanks. To prepare the SB (O+V)_Ctrl and SB (O+V)_AS treatments, 800 mL of filtered water + 500 mL of shoal water (retrieved from the corresponding demo tank) were placed in the test tank. To prepare the demo tank (containing a mixed sex shoal of 4 females and 4 males zebrafish as for the case of these treatments) 500 mL of filtered water were added – this was done to re-establish the final volume of 1.3 L after the removal of the 500 mL of shoal water that were introduced into the test tank. The used 500 mL volume of shoal water was established to minimize the crowding stress induced to the shoal when lowering the water level[5] (the same holds for experiment II). Shoal assembly in the demo tank was done 2 days before testing (the same holds for experiments II and III), so that focal fish would have visual contact with a stable shoal with established hierarchies (after 3 hours of shoaling in a tank, zebrafish already exhibit distinct territories[4]). Focal fish had visual contact with the demo tank overnight to promote habituation and contribute to create a baseline effect. On the following day, all focal fish were tested and behaviour video recorded.

Experiment II. A total of 80 focal naïve male zebrafish were used (20 per treatment). Each focal fish was submitted to a single test corresponding to one of four possible treatments (see main Fig. 3A): alone focal fish administered with AS (Alone_AS); focal fish administered with AS and exposed to shoal water [SB (O)_AS]; focal fish administered with AS and exposed to a shoal of 8 conspecifics [SB (V)_AS]; and focal fish administered with AS and exposed simultaneously to shoal water and a shoal of 8 conspecifics [SB (O+V)_AS]. As in experiment I, we were able to

test eight focal fish per day (two fish per treatment). On the afternoon of the day before the test, fish were randomly removed from their stock tanks and isolated in each test tank overnight. Per session, the order of the treatments attributed to each tank was done in a randomized fashion. To prepare the Alone_AS treatment, 1.3 L of filtered water were placed both in the test and demo tanks. In the SB (O)_AS treatment, 800 mL of filtered water + 500 mL of shoal water (retrieved from a demo tank attributed to a [SB (V)_AS] treatment) were placed in the test tank and the demo tank was filled with 1.3 L of filtered water. In the SB (V)_AS treatment, 1.3 L of filtered water were placed in the test tank and 500 mL of filtered water were added to the demo tank (containing a mixed sex shoal of 4 females and 4 males zebrafish) to re-establish the final volume of 1.3 L after the removal of the 500 mL of shoal water that were included in the test tank of the SB (O)_AS treatment. In the SB (O+V)_AS treatment, 800 mL of filtered water + 500 mL of shoal water (retrieved from the corresponding demo tank) were placed in the test tank, and 500 mL of filtered water were added to the demo tank (containing a mixed sex shoal of 4 females and 4 males zebrafish) to re-establish the final volume of 1.3 L after the removal of the 500 mL of shoal water that were transferred to the test tank. Focal fish had visual contact with the demo tank overnight to promote habituation and contribute to create the baseline effect. On the following day, all focal fish were tested and behaviour video recorded.

Experiment III. A total of 160 focal naïve male zebrafish were used (20 per treatment). Each focal fish was submitted to a single test corresponding to one of eight possible treatments (see main Fig. 4A): alone focal fish administered with water (Alone_Ctrl); alone focal fish administered with AS (Alone_AS); focal fish administered with water and exposed to a shoal of 2 conspecifics [SB (2)_Ctrl]; focal fish administered with AS and exposed to a shoal of 2 conspecifics [SB (2)_AS]; focal fish administered with water and exposed to a shoal of 4 conspecifics [SB (4)_Ctrl]; focal fish administered with AS and exposed to a shoal of 4 conspecifics [SB (4)_AS]; focal fish administered with water and exposed to a shoal of 8 conspecifics [SB (8)_Ctrl]; and focal fish administered with AS and exposed to a shoal of 8 conspecifics [SB (8)_AS]. In the case of this experiment, shoals were defined as groups of 2 to 8 conspecifics, which is a fluctuation in terms of group size that is within the variation reported (2-10 and 2-30, as seen in [6] and [7], respectively) for the number of fish integrating a shoal in the wild. We were able to test eight focal fish per day (one fish per treatment). On the afternoon of the day before the test, fish were randomly removed from their stock tanks and isolated in each test tank overnight. Per session, the order of the treatments attributed to each tank was done in a randomized fashion. To prepare the Alone_Ctrl and Alone_AS treatments, 1.3 L of filtered water were placed both in the test and demo tanks. To assemble the [SB (2)_Ctrl] and [SB (2)_AS] treatments, 1.3 L of filtered water were placed in the test tank. In these treatments, the demo tanks contained a mixed sex shoal of 1 female and 1 male zebrafish. To prepare the [SB (4)_Ctrl] and [SB (4)_AS] treatments, 1.3 L of filtered water were placed in the test tank. In the case of these treatments, the demo tanks contained a mixed sex shoal of 2 females and 2 males zebrafish. To prepare the [SB (8)_Ctrl] and [SB (8)_AS] treatments, 1.3 L of filtered water were placed in the test tank. In the case of these treatments, the demo tanks contained a mixed sex shoal of 4 females and 4 males zebrafish. Regarding the demo tanks containing shoals, 500 mL of shoal water were removed and re-established by 500 mL of filtered water – this was done to replicate the shoal water removal of previous experiments (I and II), as to have all demo tanks with shoals under the same experimental conditions. Focal fish had visual contact with the demo tank during the overnight period to contribute to create the baseline effect.

Behavioural trials. In all experiments, 5 min (baseline) after video recording was initiated, 0.754 mL of filtered water (control treatments) or 0.754 mL of AS (treatments with AS) were delivered to the test tank through the PVC tubing with the help of a 10 mL Terumo® syringe. The AS aliquot was thawed before the trials and kept on ice throughout the entire experimental session to prevent AS degradation. The test lasted for 30 min, after which each focal fish was immediately euthanized with an overdose of tricaine solution (MS222, Pharmaq; 500-1000 mg/L). Gender was confirmed by visual inspection of the gonads. Test and demo tanks were sprayed with 70% ethanol and rinsed with filtered water between treatments, to eliminate hormone and odour residues. All test trials were conducted between 10:30 a.m. and 07:30 p.m. and the different experimental groups were intermixed throughout the day to account for possible diurnal variations in behaviour. A gap of 2.30 hours between lights onset and the start of the behavioural trials was used to prevent possible confounding effects of spawning

behaviour that may have occurred in the shoals (since zebrafish are crepuscular breeders with a peak of mating behaviour at dawn[8,9]).

AS extraction. AS was extracted using a modified version of the protocol described in [10]. Ten-month-old donor zebrafish (8 females and 8 males) were used to prepare all three sets of AS (one for each experiment). The total number, sex-ratio and age of individuals used, allowed to control for possible variations in AS content (see Supplementary Fig. 2F-G for AS efficiency across experiments). Fish were retrieved from their raising tanks and placed in a small tank to proceed with AS extraction. Fish were collected from the small tank, rinsed with distilled water and excess of water was removed from skin with a paper towel. Fish were then quickly sacrificed by breaking the spinal cord with tweezers. Fish were fixed in a silicon bed and fifteen (fourteen vertically and one horizontally) shallow cuts were made on each side of the trunk with the help of a surgical scalpel blade. Fish were placed in a petri dish and 50 mL of distilled water (25 mL on each side) were used to wash the cuts (a 20 mL Terumo® syringe without the needle was used to perform the washing). Superficial cuts are sufficient to extract AS, as AS is known to be released upon skin damage[10]. There was no blood contamination in the AS solution. The solution was then passed through filter paper to avoid solution contamination with residues. The same process was repeated for all 16 fish used. In the end, the AS solution was filtered a second time. The 800 mL of solution were divided into 10 mL aliquots and stored at -20ºC until further use. During the collection process, the solution was kept on ice. AS extraction was always done at least one day before experiments initiation so that all aliquots would pass through the thawing process. The same solution of AS was not used across different experiments to prevent possible degradation.

Behavioural analysis. For each focal fish, 2 videos were analyzed corresponding to the side and top views respectively (see Supplementary Fig. 1). The extracted x, y, z coordinates were subsequently analyzed using XYZ2B – Fish XYZ to Behaviour (https://github.com/joseaccruz/xyz2b), a custom-made code that combines a set of python scripts and infers erratic movement and freezing behaviour from x, y, z data produced by the EthoVision. Erratic movement is a complex behaviour characterized by sharp changes in direction or velocity, commonly associated with fast acceleration bouts and stochastic turns[11]. Therefore, the xyz2b decides on erratic movement behaviour if fish acceleration > 8 m/s^2. Freezing behaviour is described as a complete cessation of movement of zebrafish (except for opercula and eyes), while at the bottom of the tank[11]. For this reason, the xyz2b decides on freezing behaviour if two conditions apply: (1) fish velocity < 0.2 m/s; and (2) fish position is inside the bottom quarter of the arena (i.e. "freezing region" - as remaining in the bottom of the tank is one of the criteria for freezing behaviour, fish position was added to avoid false freezing computations when zebrafish is hovering[11], typically near the water surface). Because of camera's perspective distortion caused by water depth, the bottom quarter part of the tank was defined as the "freezing region", to include in the image the cases where fish exhibit freezing behaviour in the most distal area from the side camera (see Supplementary Fig. 1A). To increase the accuracy of xyz2b, erratic movement and freezing behaviours were only inferred when side and top views information regarding acceleration and velocity was in accordance (e.g. erratic movement was only considered when both side and top views data indicated acceleration > 8 m/s^2 and freezing was only considered when both side and top views data indicated velocity < 0.2 m/s). The total duration of erratic movement and freezing was calculated over 300 sec bins (5 min) and presented in percentage of total time. In the cases where 10 min bins are presented, data from the two corresponding 5 min bins was summed. The xyz2b generates a 3D plot (see Fig. 2B in main text) representative of fish behaviour, in which the red dot area is proportional to freezing time and the grey line is representative of the remaining time in swimming behaviour. In order to test SB in zebrafish using AS as a fearful event, we established the behavioural parameter (erratic movement or freezing) that better describes zebrafish response to this stimulus, using as reference the response to AS when a fish is alone. Since in the case of our behavioural treatments we were exploring possible variations in fear behaviour due to absence or presence of conspecifics, we decided that the criteria for choosing the behavioural parameter were: 1) frequency of the behaviour - in terms of percentage of time; 2) consistency of the behaviour in time. Thus, the behaviour that was both frequent and

consistent in time was the representative parameter of alarm reaction in our study – in this case, freezing.

Brain microdissection protocol. Only fish from the treatments Alone_Ctrl, Alone_AS, SB (8)_Ctrl and SB (8)_AS of experiment III were subjected to the brain microdissection protocol. Twelve fish out of the 20 fish used for the behavioural experiments were selected. Individuals closer to the mean value of their respective treatment were chosen since they were considered the best representatives of the average population's response for each behavioural treatment. Zebrafish embedded heads were retrieved from -80°C and sliced on a cryostat (Leica CM 3050 S) set at -22°C, in serial 150µm-thick sections in the coronal plane, thaw mounted in regular glass slides and refrozen in the cryostat. Sections were sequentially collected from the point where olfactory bulbs were visible until the tectal ventricles were evident. Once all sections of interest were sampled, they were microdissected under a stereoscopic microscope (Nikon SMZ745) on top of a cold plate. Brain nuclei were identified and classified as in [1] and harvested with a modified 27G needle (inner diameter = 210µm). To minimize the risk of cross-contamination a single needle was used per brain nucleus, and a new set of needles was used for each fish. To prevent RNA degradation, all needles were cleaned with RNaseZAP™ (Sigma-Aldrich, Hamburg, Germany), followed by 70% ethanol, and dried overnight in an incubator (VWR INCU-Line® IL10) at 70°C. We collected tissue from candidate brain areas (see main text for mammalian homologies and function): medial zone of the dorsal telencephalic area (Dm); supracommissural nucleus of the ventral telencephalic area (Vs); preoptic area (POA) and ventral nucleus of the ventral telencephalic area (Vv). Dm was sampled from the first slice where Vv was sampled (Supplementary Fig. 3A) until the last slice where the parvocellular preoptic nucleus, anterior part (PPa) appears (Supplementary Fig. 3F). Vv was sampled from the last slice where the olfactory bulbs appear (Supplementary Fig. 3A) until the slice immediately before the appearance of the diencephalic ventricle (DiV; Supplementary Fig. 3D). Vs was punched only once in the first slice where the DiV appears (Supplementary Fig. 3D). POA was collected from the first slice where the DiV appears (Supplementary Fig. 3D) until the last slice where the PPa appears (Supplementary Fig. 3F). All brain nuclei were sampled in both hemispheres at once (see Supplementary Fig. 3). The harvested tissue was immediately injected with a 10 mL syringe into a 1,5 mL autoclaved microtube filled with 50 µL QIAzol Lysis Reagent (Qiagen). Except for Vs, more than one punch was harvested for each brain nucleus (see Supplementary Fig. 3). Punches from the same brain nucleus were pooled into the same microtube. Samples were kept on ice and covered with aluminium foil until all slides were processed and stored at -80°C until RNA extraction.

Gene expression analysis

RNA extraction and DNA Synthesis. RNA extraction was performed with RNeasy® Lipid Tissue Mini Kit using the manufacturer protocol with minor modifications. RNA integrity was measured in 10% of all samples processed (randomly chosen) using 2100 Bioanalyzer (Agilent Technologies). Samples were stored at -80°C until cDNA synthesis. iScript™ cDNA Synthesis Kit (Bio-Rad) was used to synthesize the DNA. Samples were incubated in a thermocycler (T100™ Thermal Cycler, Bio-Rad) in accordance with manufacturer's instructions (5min at 25°C, 60min at 42°C and 5min at 85°C) and stored at -20°C.

Primer design-optimization-efficiency and normalization to the reference gene. Primers were designed using Primer3[12,13] following standard parameters with some changes (changes are presented in parenthesis): primer size (Min:18, Opt:20, Max:22); primer Tm (Min:57.0, Opt:60.0, Max:63.0); max Tm difference (3.0); primer GC% (Min:45.0, Opt:50.0, Max:60.0); product size ranges (100-200); and max GC in primer 3' end (3). Using the Quick Primer Test tool of the FastPCR v5.4[14,15], primers presenting the minimum number of primer dimers at maximum sensitivity and quality≥80 were selected. Thereafter, Primer-BLAST[16] was used to confirm if the selected primers were specifically amplifying the target gene, without amplifying any other gene in the *Danio rerio* genome. Primers were commercially synthesized (Sigma-Aldrich, Hamburg, Germany). To ascertain the optimal annealing temperature, a PCR was

conducted (T100™ Thermal Cycler, Bio-Rad) with a gradient of temperatures. Moreover, to ascertain if primers were amplifying the right sequences, PCR products were sequenced. Primers efficiency was optimized through a two-fold dilution series, in order to guarantee an accurate quantification regardless of the DNA template concentration. Target gene expression was normalized to the reference gene through the equation: $2^{Ct_{Ref}-Ct_{Target}}$, where Ct_{Ref} is the cycle threshold for the reference gene and Ct_{Target} is the cycle threshold for the target gene. Therefore, target gene expression is represented as its relative expression to the reference gene. Mean of the Cts of the three technical replicates were used. A technical replicate was discarded whenever its Ct was 0.5 below or above the mean Ct of the replicates. A sample did not undergo further runs if: 1) at least two of the three technical replicates were not discarded; 2) in the case that only two replicates remained, the absolute value of the difference between them was less than 0.5. Samples that did not fulfil these parameters were repeated until three times. Lastly, if after these three runs the parameters were not fulfilled, the sample was discarded and considered as a missing value.

Real-time PCR (qPCR). qPCR was used to determine mRNA expression levels of an immediate early gene (*c-fos)* and a reference gene (*18s rRNA). c-fos* is a transient marker of neuronal activity and its expression has been used to characterize brain activation in response to behavioural manipulations[2]. qPCR assays were performed using SYBR® Green PCR Master Mix (Applied Biosystems™, Thermo Fisher). Each reaction mix consisted of: 1.7 µL nuclease-free water, 4 µL of SYBR Green, and 0.15 µL of each primer (with a concentration of 50 pmol/µL). Three technical replicates were performed for each sample, all run in the same plate. Quantification was measured on a QuantStudio™ 7 Flex Real-Time PCR System (Applied Biosystems™, Thermo Fisher) using the following reaction protocol: (i) denaturation (5 min at 95°C); (ii) amplification and quantification (40 cycles; 30 s at 95°C, primer-specific annealing time and temperature, 30 s at 72°C with a single fluorescence measurement); and (iii) melting curve assessment (30 s at 95°C; 30 s at 55°C, followed by an increase from 55–95°C with a heating rate of 0.05°C/s and a continuous fluorescence measurement; 30s at 95°C).

Statistical Analysis

Behaviour and relative gene expression statistical analysis were performed on the statistical software packages STATISTICA v. 10 (StatSoft, Inc.) and SPSS® Statistics v. 21 (IBM). Normality and homogeneity of variance of the data were tested, and the appropriate statistics were used as required.

Behaviour and relative gene expression statistical analysis. All behavioural statistical analysis followed the same approach. Repeated measures ANOVA were performed – with treatment as categorical predictor and freezing in each time bin (5 or 10 min) as dependent variable – to compare freezing between: 1) baseline and the 1st 5 min after AS administration; 2) 10 min bins over the 30 min test. When sphericity was not assumed, Repeated measures ANOVA with Greenhouse-Geisser correction (SPSS) was used. Differences in relative gene expression between treatments were assessed using one-way ANOVA with treatment as categorical predictor and gene expression in Dm, Vv, Vs and POA as dependent variables. One-way ANOVA and repeated measures ANOVA were followed by LSD posthoc. All pairwise comparisons extracted from the LSD posthoc matrix were corrected for multiple comparisons using the sequential Bonferroni correction (see the explanation of the method below).

Sequential Bonferroni correction. Both for behavioural and relative gene expression analysis, sequential Bonferroni corrections were performed for all pairwise comparisons to correct for multiple comparisons and corrected p values (p') reported. Statistical significance was set at adjusted p-value - $p' < 0.05$. Uncorrected p-values were first rank-ordered by significance (from the smallest to the highest p-value) and then corrected using the following algorithm (for m = number of hypothesis tested, i = hypothesis number, j = ranked position of p-value):

$p'_{(i)} = \max_{j \leq i}\{(m - j + 1)p_{(j)}\}_1$, where $\{x\}_1 \equiv \min(x,1)$

The correction procedure was applied until the uncorrected p-value stopped being significant, $p > 0.05$.

Brain network analysis. Brain co-activation of the different treatments was assessed as in [2]. Pearson product moment correlations were computed between *c-fos* expression in each pair of brain nuclei, for each behavioural treatment. These correlations were considered as indicative of co-activation between brain areas, in that positive correlations correspond to phasic activity and negative correlations to out-of-phase activity. The occurrence of different patterns of brain co-activation associated with different behavioural treatments was assessed by testing the association between any two Pearson correlation matrices using the quadratic assignment procedure (QAP) correlation test with 5000 permutations[2]. The null hypothesis in QAP is that there is no association between matrices[17]. Therefore, correlation matrices were considered different when QAP p-value was higher than 0.05. Finally, cohesion and centrality network measures (density and eigenvector centrality, respectively) were used to structurally characterize the brain network underlying each treatment. Density – the proportion of all possible connections that are present in a brain network – was used as a measure of cohesion[18]. As to compare the density of connections among different behavioural treatments (differences in the mean strengths of the relation between two brain areas), we used a bootstrap t-test approach with 5000 sub-samples. Eigenvector centrality, here used as a measure of centrality, considers the number of direct connections that a brain area has and how well connected its relationships are[18]. Network statistical analyses were performed using UCINET 6[18]. Brain nuclei co-activation network figures (Fig. 4D) were produced using a custom-made python code.

References

1. Wullimann, M. F., Rupp, B. & Reichert, H. *Neuroanatomy of the Zebrafish Brain: A Topological Atlas*. (Birkhäuser Basel, 1996). doi:10.1007/978-3-0348-8979-7

2. Teles, M. C., Almeida, O., Lopes, J. S. & Oliveira, R. F. Social interactions elicit rapid shifts in functional connectivity in the social decision-making network of zebrafish. *Proc. R. Soc. B Biol. Sci.* **282,** 20151099 (2015).

3. Pavlidis, M. *et al.* Husbandry of zebrafish, Danio rerio, and the cortisol stress response. *Zebrafish* **10,** 524–31 (2013).

4. Pérez-Escudero, A., Vicente-Page, J., Hinz, R. C., Arganda, S. & de Polavieja, G. G. idTracker: tracking individuals in a group by automatic identification of unmarked animals. *Nat. Methods* **11,** 743–748 (2014).

5. Ramsay, J. M. *et al.* Whole-body cortisol is an indicator of crowding stress in adult zebrafish, Danio rerio. *Aquaculture* **258,** 565–574 (2006).

6. Pritchard, V., Lawrence, J., Butlin, R. K. & Krause, J. Shoal choice in zebrafish, Danio rerio: the influence of shoal size and activity. *Anim. Behav.* **62,** 1085–1088 (2001).

7. Spence, R., Jordan, W. C. & Smith, C. Genetic analysis of male reproductive success in relation to density in the zebrafish, Danio rerio. *Front. Zool.* **3,** 5 (2006).

8. Spence, R. *et al.* The distribution and habitat preferences of the zebrafish in Bangladesh. *J. Fish Biol.* **69,** 1435–1448 (2006).

9. Engeszer, R. E., Patterson, L. B., Rao, A. a & Parichy, D. M. Zebrafish in the wild: a review of natural history and new notes from the field. *Zebrafish* **4,** 21–40 (2007).

10. Speedie, N. & Gerlai, R. Alarm substance induced behavioral responses in zebrafish (Danio rerio). *Behav. Brain Res.* **188,** 168–177 (2008).

11. Kalueff, A. V *et al.* Towards a comprehensive catalog of zebrafish behavior 1.0 and beyond. *Zebrafish* **10,** 70–86 (2013).

12. Koressaar, T. & Remm, M. Enhancements and modifications of primer design program Primer3. *Bioinformatics* **23,** 1289–91 (2007).

13. Untergasser, A. *et al.* Primer3--new capabilities and interfaces. *Nucleic Acids Res.* **40,** e115 (2012).

14. Kalendar, R., Lee, D. & Schulman, A. H. Java web tools for PCR, in silico PCR, and oligonucleotide assembly and analysis. *Genomics* **98,** 137–44 (2011).

15. Kalendar, R., Lee, D. & Schulman, A. H. in *DNA Cloning and Assembly Methods, Methods in Molecular Biology* (eds. Valla, S. & Lale, R.) 271–302 (Humana Press, 2014).

16. Ye, J. *et al.* Primer-BLAST: A tool to design target-specific primers for polymerase chain reaction. *BMC Bioinformatics* **13,** 134 (2012).

17. Borgatti, S. P., Everett, M. G. & Johnson, J. C. *Analyzing Social Networks*. (SAGE Publications Limited, 2013).

18. Borgatti, S. P., Everett, M. G. & Freeman, L. C. Ucinet for Windows: Software for social network analysis. *Anal. Technol.* (2002).
